# Lumbar intramedullary spinal cord metastasis from esophageal cancer treated with palliative radiotherapy: a case report

**DOI:** 10.1007/s13691-025-00819-1

**Published:** 2025-10-19

**Authors:** Nobuko Utsumi, Hidemasa Kawamura, Takafumi Yamano, Toyokazu Hayakawa, Norifumi Mizuno, Shuichi Ueno, Mio Saito, Fumiharu Machida, Yusuke Matsumoto, Mariko Umeda, Yuito Kato, Youichi Kumagai, Takeo Takahashi

**Affiliations:** 1 Department of Radiation Oncology, Saitama Medical Center, Saitama Medical University, 1981, Kamoda, Kawagoe, Saitama 350-8550 Japan; 2 Department of Digestive Tract and General Surgery, Saitama Medical Center, Saitama Medical University, 1981, Kamoda, Kawagoe, Saitama 350-8550 Japan

**Keywords:** Intramedullary spinal cord metastasis, Esophageal cancer, Palliative radiotherapy

## Abstract

Intramedullary spinal cord metastasis (ISCM) is a rare condition. ISCM from esophageal cancer is extremely uncommon. We report a rare case of lumbar ISCM from esophageal cancer in a woman in her 70 s, initially diagnosed with clinical stage IVA (cT4bN1M0) esophageal cancer. She underwent definitive radiotherapy (60 Gy in 30 fractions) with concurrent chemotherapy of 5-fluorouracil and cisplatin (FP), followed by two additional cycles of FP chemotherapy. A complete response was maintained for 1 year and 7 months post-radiotherapy by endoscopy. However, the patient began to experience mild bladder and rectal dysfunction and back pain. 18F-fluorodeoxyglucose positron emission tomography/computed tomography (FDG-PET/CT) revealed high FDG uptake in the spinal cord cavity at the L1 level and contrast-enhanced spinal magnetic resonance imaging (MRI) showed an intramedullary tumor corresponding to the areas of FDG accumulation. Based on the appearance of new lesions and elevated tumor markers, the lesion was diagnosed as ISCM from esophageal cancer rather than a primary spinal cord tumor, and palliative radiotherapy (20 Gy in 5 fractions) was promptly administered. Two months after radiotherapy, the patient’s neurologic symptoms improved, and she continued treatment with immune chemotherapy. To our knowledge, this is the first reported case of immune checkpoint inhibitors after the diagnosis of ISCM from esophageal cancer. Although rare, ISCM should be considered in cancer patients presenting with new neurological symptoms, and timely multidisciplinary intervention is essential for optimal management.

## Introduction

Intramedullary spinal cord metastasis (ISCM) is a rare condition, accounting for only 0.9–2.1% of cancer patients in autopsy series [1, 2], in contrast to brain metastases which occur in approximately 20% of cases [3]. More than half of ISCMs originate from lung cancer [4]. To the best of our knowledge, only a few cases of ISCM originating from esophageal cancer have been reported in the literature [5, 6]. Here, we report a rare case of lumbar ISCM from esophageal cancer, diagnosed 1 year and 7 months after definitive chemoradiotherapy.

## Case report

**Fig. 1:**
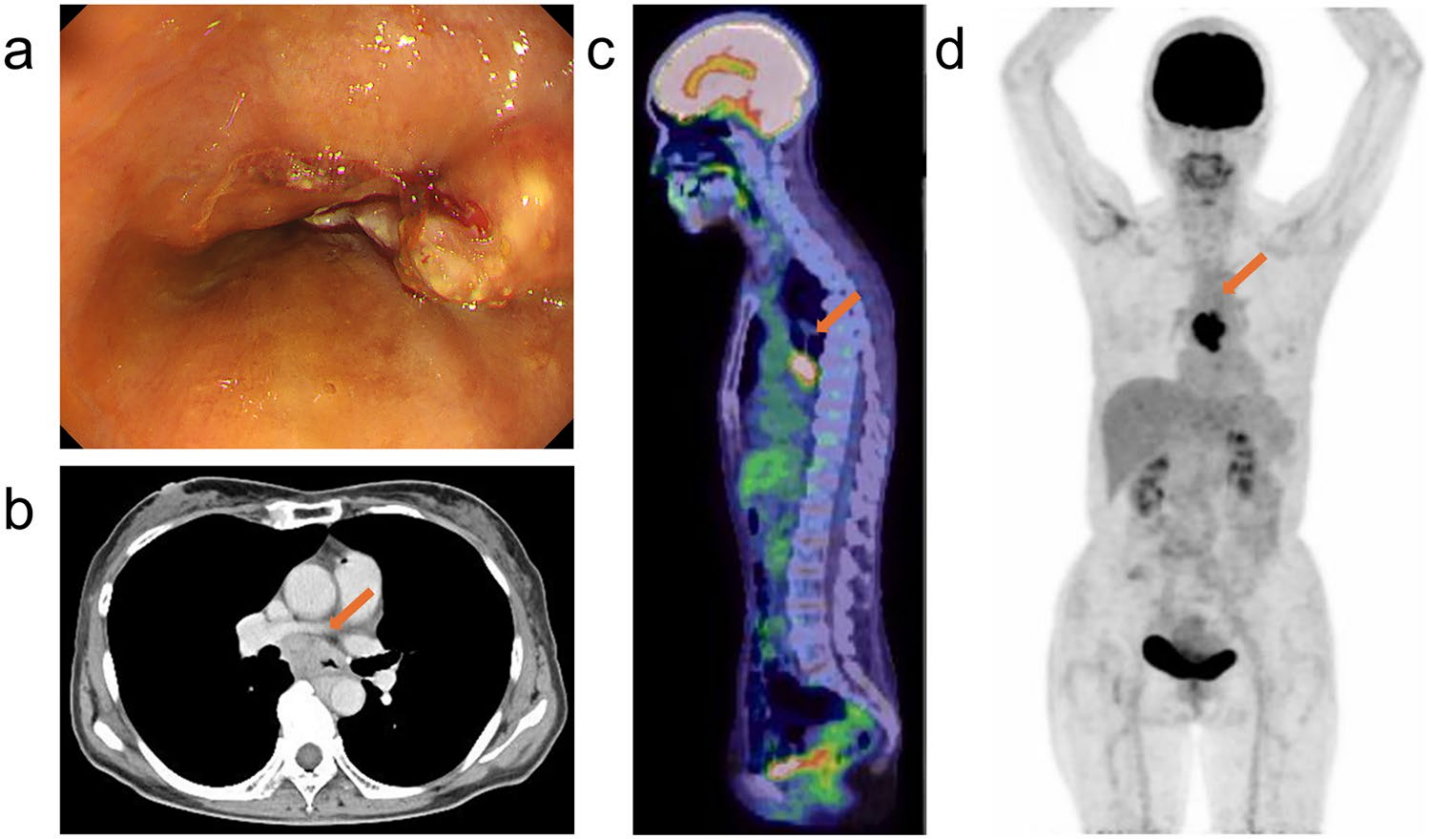
**a** Upper gastrointestinal endoscopy showed type 1 esophageal cancer in the middle thoracic esophagus. **b** Iodine contrast CT showed esophageal cancer invading the aorta (indicated by arrow in **b**; axial CT image). **c and d** FDG-PET/CT showed accumulation in the primary lesion with mediastinal lymphadenopathies (indicated by arrows in **c**; sagittal fused PET-CT scan and **d**; MIP image from PET scan) and no distant metastases. *CT* computed tomography, *FDG-PET* 18F-fluorodeoxyglucose positron emission tomography, *MIP* maximum intensity projection

**Fig. 2:**
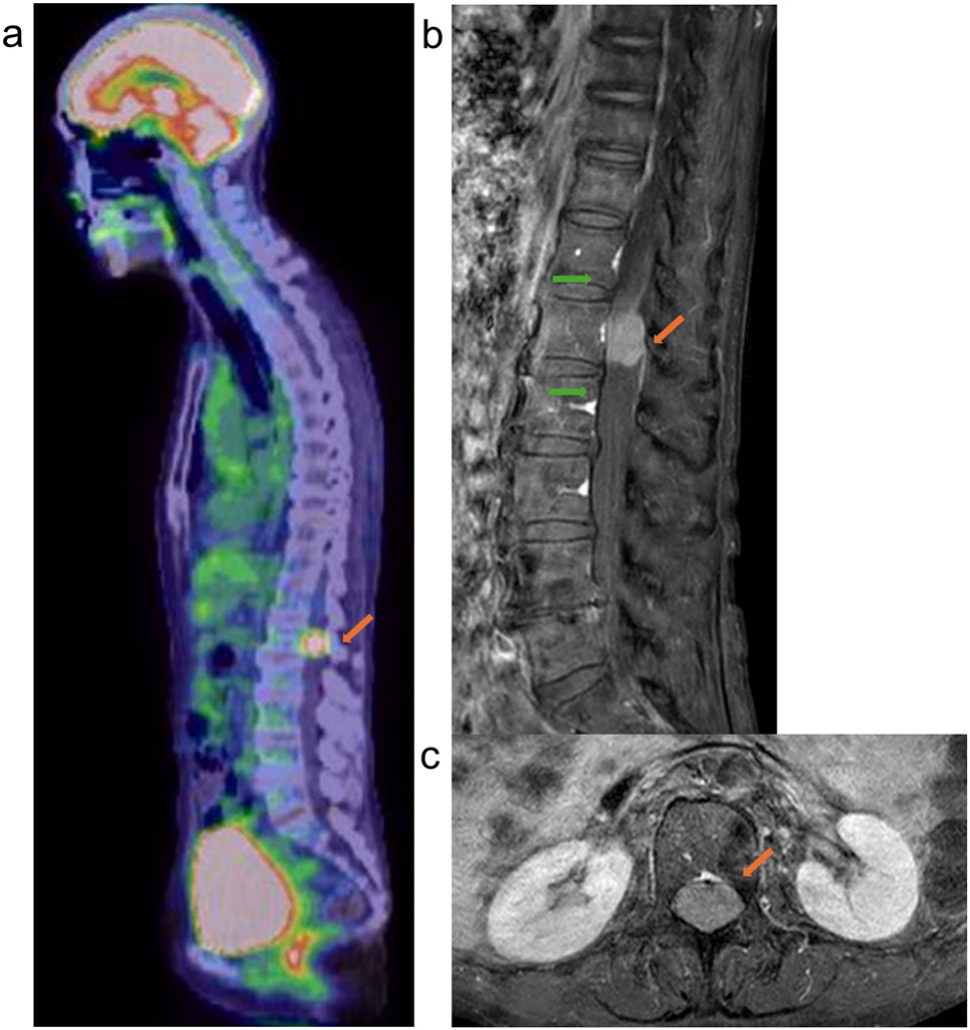
**a** FDG-PET/CT showed accumulation in the spinal cord cavity at L1 level (indicated by arrow in **a**; sagittal fused PET–CT scan). **b and c** Post-gadolinium T1-weighted MRI images showed a relatively uniform enhanced tumor in the spinal cord cavity at L1 level (indicated by orange arrows in **b**; sagittal MRI and **c**; axial MRI). The flame sign was observed (indicated by green arrows in **b**). *FDG-PET/CT* 18F-fluorodeoxyglucose positron emission tomography/computed tomography, *MRI* magnetic resonance image

**Fig. 3:**
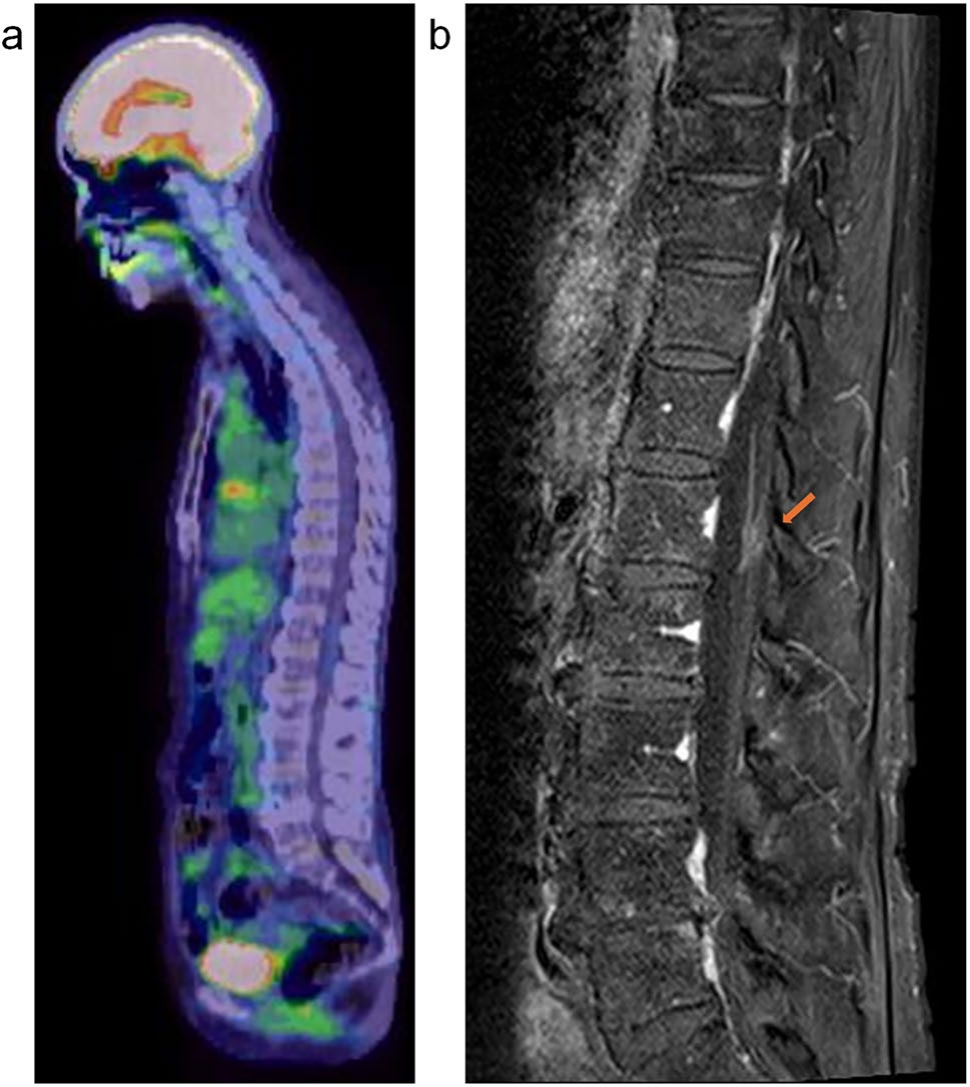
**a** FDG-PET/CT taken 4 months after radiotherapy showed that FDG accumulation in the ISCM had almost disappeared. **b** Post-gadolinium T1-weighted MRI images showed a marked shrinkage of ISCM (indicated by arrow in (**b**); sagittal MRI). *FDG-PET/CT* 18F-fluorodeoxyglucose positron emission tomography/computed tomography; *MRI* magnetic resonance image

A female patient in her 70 s with no history of cancer presented with dysphagia. Upper gastrointestinal endoscopy revealed a suspected type 1 esophageal cancer in the middle thoracic region (Fig. 1a). Histological examination confirmed squamous cell carcinoma of the esophagus. Whole-body computed tomography (CT) and 18F-fluorodeoxyglucose (FDG) positron emission tomography (PET)/CT scans showed tumor invasion of the aorta and mediastinal lymphadenopathy, but no distant metastases (Fig. 1b, c and d). The clinical diagnosis was stage cT4bN1M0, stage IVA esophageal cancer, according to the 8th edition of the Union for International Cancer Control staging system. The patient underwent definitive radiotherapy with a total dose of 60 Gy in 30 fractions with concurrent chemotherapy of 5-fluorouracil and cisplatin (FP). Prophylactic lymph node area was included in the irradiation field for a total dose of 40 Gy, after which irradiation was continued to the primary esophageal cancer and involved lymph nodes to a total dose of 60 Gy, with the spinal cord excluded from the irradiation field. Acute adverse events included esophagitis, anemia, thrombocytopenia, and dermatitis, each of which was grade 1. After completion of radiotherapy, two additional courses of FP chemotherapy were administered. One month after radiotherapy, complete response (CR) was confirmed by CT and endoscopy with no evidence of residual cancer using biopsy histology. The CR status was maintained for 1 year and 7 months post-radiotherapy, confirmed by follow-up examinations. Tumor markers squamous cell carcinoma antigen (SCC) was maintained below 1 ng/ml after radiotherapy, and anti-p53 antibody decreased promptly from 45 U/ml at the end of radiotherapy to 6.34 U/ml in 9 months. However, in 1 year and 7 months post-radiotherapy, both increased (SCC; 2.1 ng/ml and anti-p53 antibody; 45.2 U/ml) and the patient began to experience mild bladder and rectal dysfunction and back pain. FDG-PET/CT revealed two lesions with high FDG uptake: one in the spinal cord cavity at the L1 level (Fig. 2a), and the other in the left side of the sacrum. Contrast-enhanced spinal magnetic resonance imaging (MRI) showed an intramedullary tumor corresponding to the areas of FDG accumulation. Post-gadolinium T1-weighted MRI demonstrated relatively homogeneous enhancement, with no abnormal findings in bones around the tumor (Fig. 2b and c). No brain metastases were observed on contrast-enhanced brain MRI. A biopsy of the spinal tumor was considered for pathological confirmation, but it was judged to be technically difficult by the orthopedic surgeons. Based on the appearance of new lesions and elevated tumor markers, and no other primary malignancies were detected, the lesion was diagnosed as ISCM from esophageal cancer rather than a primary spinal cord tumor. Also, MRI findings of the spine outside the lumbar region and of the brain supported the less possibility of intrathecal dissemination. A urethral balloon catheter was placed, and palliative radiotherapy (20 Gy in 5 fractions) to both lumbar ISCM and bone metastases of left side of the sacrum was promptly administered. Two weeks later, immune chemotherapy with FP and pembrolizumab was initiated. No acute adverse events were seen. The numeric rating scale (NRS) pain score was recorded as 8 at the onset of radiotherapy and decreased to nearly 0 by the end of treatment, although pain medication was still administered. Two months after radiotherapy, the urethral balloon catheter was successfully removed. At 3 months post-radiotherapy, pain medication was discontinued and the NRS score remained at 0, whereas numbness from the hip to the leg persisted. The patient continued treatment with immune checkpoint inhibitors. FDG-PET/CT performed 4 months after radiotherapy demonstrated that FDG uptake in the ISCM had almost disappeared (Fig. 3a), and uptake in the bone metastases had also markedly decreased. MRI revealed a significant reduction in the size of the ISCM (Fig. 3b).

## Discussion

ISCM is an uncommon condition. Clinically, ISCM affects only 0.1–0.4% of all cancer patients [4], and merely 5% of these cases are diagnosed by physicians before death [1]. ISCM accounts for 4.2–8.5% of all central nervous system metastases and 1–3% of all intramedullary tumors [4, 7–9]. According to autopsy studies, ISCM is found in only 0.9–2.1% of cancer patients, whereas brain metastases are present in approximately 20% [5]. Although rare, ISCM can cause severe neurological symptoms, which can be fatal. Dam-Hieu et al. evaluated neurological function in 19 patients with ISCM, revealing that 12 patients (63%) were non-ambulatory, while 6 (31%) had mild functional impairments. The mortality rate is approximately 80% within 3 to 4 months of symptom onset [10], and the overall median survival is reported to be 4–6 months [11, 12]. Lung cancer is the most common primary site for ISCM, accounting for 64% of cases, followed by breast cancer (11%), malignant melanoma (5%), renal cell carcinoma (4%), colorectal cancer (3%), and malignant lymphoma (3%) [13]. Reports of ISCM originating from esophageal cancer are extremely rare.

According to a review of the literature, four case reports of ISCM originating from esophageal cancer have been identified [5, 10, 11, 14] (Table 1). The reported symptoms were severe, and all patients died within 20 days to 3 months. In our case, the ISCM was located at the L1 level of the lumbar spinal cord, which is a lower spinal level than in the previously reported cases. As a result, the symptoms were less severe, making systemic treatment with immune chemotherapy feasible.

**Table 1:** A literature review of 5 available reported cases of ISCM from esophageal cancer, including the present report

First author (year)	Age (years)/gender	Time since primary cancer diagnosis	Location	Presentation	Other metastases	Treatments	Follow-up/outcome
Dam-Hieu (2009)	61/male	1 month	C4	Quadriparesis; sphincter disorders	Brain	Surgery; RT	3 months/died
Nakamura (2014)	78/male	2 months	C2	Diplegia; respiratory failure	Lymph nodes	PRT	20 days/died
Grillo (2019)	35/female	1 year	Th9	Paraplegia; neurogenic bladder	Lymph nodes; vertebral bone; brain	PRT	2 months/died
Shen (2023)	68/male	2 years	C4-5	Neck pain; right hemiplegia; dysuria; dyspnea	Lung; liver; brain	BSC	1 month/died
Utsumi (This report)	71/female	1 year 9 months	L1	Bladder and rectal dysfunction; back pain	Sacrum	PRT; immune chemotherapy	2 months/improved

*RT* radiotherapy, *PRT* palliative radiotherapy, *BSC* best supportive care

With advancements in cancer therapies and improvements in diagnostic imaging techniques such as contrast-enhanced MRI, the number of patients diagnosed with ISCMs is expected to increase. Indeed, recent studies have shown a rising number of ISCM cases [4, 15]. Early diagnosis of ISCM is crucial to prevent the progression of spinal cord-related symptoms. Two characteristic post-gadolinium MRI features of ISCMs have been reported: the rim sign and the flame sign [16]. The rim sign is defined as a complete or partial thin peripheral rim of gadolinium enhancement that is more intense than the central enhancement of a non-cystic/necrotic lesion. The flame sign is described as an ill-defined, flame-shaped region of gadolinium enhancement at the superior and/or inferior margin of an otherwise well-defined lesion. Rykken et al. analyzed 64 ISCMs and 64 primary spinal cord tumors and found that the rim and flame signs, individually and in combination, were significantly more common in ISCMs than in primary intramedullary tumors (*p* < 0.0001 for each) [16]. In our case, MRI showed relatively homogeneous gadolinium enhancement of the lesion, making the rim sign equivocal. However, the flame sign was observed (Fig. 2b). They also reported a correlation between these signs and the size of ISCM. In their analysis, the mean lesion size was 31 mm versus 14 mm for ISCMs with and without the rim sign, and 34 mm versus 14 mm for those with and without the flame sign. In the present case, the size of the ISCM was 19 mm, and its relatively small size may explain the absence of the rim sign.

The optimal management of patients with ISCM remains challenging due to the wide variety of clinical cancer stage and the lack of controlled studies evaluating various therapeutic options. A retrospective analysis of 19 patients with ISCM, which included only one case of esophageal cancer, reported that microsurgical excision was performed in 13 patients (68%), with complete tumor resection achieved in 11 and incomplete resection in two[11]. Median survival was significantly longer in surgically treated patients compared to those receiving conservative treatment (7.4 months vs. 2.6 months, *p* < 0.03) [11]. Although surgery may be considered the optimal therapeutic approach in selected cases [17–19], it is not always indicated—particularly when the primary tumor is uncontrolled or the patient’s general condition is poor. Radiotherapy remains an established, minimally invasive treatment option for local tumor control. A literature review of 293 ISCM cases indicated that conservative management with radio- and chemotherapy is the most popular treatment choice [12]. It is important to initiate treatment promptly in patients presenting with spinal cord symptoms. In our case, radiotherapy was started the day after the initial visit to the Radiation Oncology Department, following a prompt discussion in multiple departments including gastrointestinal surgery, orthopedic surgery, and radiation oncology. Fortunately, the patient’s neurological symptoms improved, enabling subsequent administration of immune chemotherapy. In a Delphi consensus study on oligometastatic oesophagogastric cancer conducted in Europe, no consensus was reached regarding the use of immune checkpoint inhibition due to insufficient data, although its use was acknowledged [20]. To our knowledge, there have been no previous reports describing the use of immune checkpoint inhibitors after the diagnosis of ISCM from esophageal cancer. As an additional examination, the Combined Positive Score (CPS), which evaluates the expression of programmed death-ligand 1, was assessed and found to be ten or greater, a cutoff value indicative of pembrolizumab efficacy. This result may explain the remarkable response to immunochemotherapy observed in the present case. Results from a subgroup analysis of Japanese participants in the KEYNOTE-590 study suggest that early tumor shrinkage and depth of response may have early and continuous on-treatment utility, respectively [21]. These findings indicate that in some patients who initially respond to immune checkpoint inhibitors, the therapeutic effect may be sustained. In the present case, a durable treatment effect is also expected. Patients with oligometastases may be good candidates for immune chemotherapy, particularly those with a high CPS. Further studies are warranted.

When the patient with esophageal cancer presents new neurological symptoms, clinicians should consider not only bone metastases but also ISCM. Thorough diagnostic evaluations are necessary; on the other hand, prompt multidisciplinary intervention at the time of diagnosis is essential to optimize patient outcomes.

In conclusion, we report a rare case of ISCM from esophageal cancer that was treated with radiotherapy followed by immune chemotherapy.

## Data Availability

The data that support the findings of this study are not openly available due to reasons of sensitivity and are available from the authors but restrictions apply to the availability of these data, which were used under license from Saitama Medical Center, Saitama Medical University for the current study, and so are not publicly available. Data are, however, available from the authors upon reasonable request and with permission from Saitama Medical Center, Saitama Medical University.
